# Antimicrobial Peptides (AMPs): Potential Therapeutic Strategy against Trypanosomiases?

**DOI:** 10.3390/biom13040599

**Published:** 2023-03-26

**Authors:** Maura Rojas-Pirela, Ulrike Kemmerling, Wilfredo Quiñones, Paul A. M. Michels, Verónica Rojas

**Affiliations:** 1 Instituto de Biología, Facultad de Ciencias, Pontificia Universidad Católica de Valparaíso, Valparaíso 2373223, Chile; mlrojas.pirela@gmail.com; 2 Instituto de Ciencias Biomédicas, Facultad de Medicina, Universidad de Chile, Santiago de Chile 8380453, Chile; ukemmerling@uchile.cl; 3 Laboratorio de Enzimología de Parásitos, Departamento de Biología, Facultad de Ciencias, Universidad de Los Andes, Mérida 5101, Venezuela; wilfredoquinone@gmail.com; 4 School of Biological Sciences, The University of Edinburgh, The King’s Buildings, Edinburgh EH9 3FL, UK; paul.michels@ed.ac.uk

**Keywords:** trypanosomiases, human sleeping sickness, Chagas disease, antimicrobial peptides, anti-Trypanosoma activity, alternative therapy

## Abstract

Trypanosomiases are a group of tropical diseases that have devastating health and socio-economic effects worldwide. In humans, these diseases are caused by the pathogenic kinetoplastids *Trypanosoma brucei*, causing African trypanosomiasis or sleeping sickness, and *Trypanosoma cruzi*, causing American trypanosomiasis or Chagas disease. Currently, these diseases lack effective treatment. This is attributed to the high toxicity and limited trypanocidal activity of registered drugs, as well as resistance development and difficulties in their administration. All this has prompted the search for new compounds that can serve as the basis for the development of treatment of these diseases. Antimicrobial peptides (AMPs) are small peptides synthesized by both prokaryotes and (unicellular and multicellular) eukaryotes, where they fulfill functions related to competition strategy with other organisms and immune defense. These AMPs can bind and induce perturbation in cell membranes, leading to permeation of molecules, alteration of morphology, disruption of cellular homeostasis, and activation of cell death. These peptides have activity against various pathogenic microorganisms, including parasitic protists. Therefore, they are being considered for new therapeutic strategies to treat some parasitic diseases. In this review, we analyze AMPs as therapeutic alternatives for the treatment of trypanosomiases, emphasizing their possible application as possible candidates for the development of future natural anti-trypanosome drugs.

## 1. Introduction

Kinetoplastids are a group of globally distributed flagellated protists which include both free-living and parasitic species responsible for serious diseases in animals and humans. These protists are distinguished by the presence of a large DNA network-containing region, known as “kinetoplast”, in their single large mitochondrion [1]. Many of the organisms that make up this group have other common characteristics such as (I) the presence of a single flagellum that originates near the kinetoplast of the mitochondrion and emanates from a pocket in the cell membrane (except for the intracellular form of *Trypanosoma cruzi*); (II) the presence of essential organelles called glycosomes, which are modified peroxisomes in which the first seven steps of glycolysis and several other metabolic processes are carried out; (III) a complex life cycle that involves multiple morphological stages with dramatic changes in their protein expression, metabolism, and membrane composition; (IV) the species-specific production of molecules that are critical for their survival and immune evasion of host; and (V) the presence of 6000 orthologous genes in common between different species that cause different diseases [2,3,4].

Within this group of organisms are included species that cause human diseases such as African trypanosomiasis (HAT or sleeping sickness), which is caused by two infective subspecies of *Trypanosoma brucei,* and Chagas disease (CD), which is caused by *T. cruzi*, both are considered Neglected Diseases by the World Health Organization (WHO) [4,5,6]. These kinetoplastid diseases affect millions of people in low- and middle-income countries, located mainly in tropical and subtropical regions, causing around 30,000 deaths per year and inducing disabling morbidities in millions more [2,5,7]. The use of drugs for the treatment of these diseases has important limitations since, in addition to many available drugs date from the early and middle of the 20th century, they have limited efficacy in advanced stages of the disease, are non-specific, and/or are highly toxic [4,7]. Additionally, in the case of CD, *T. cruzi* can adopt quiescent and phenotypically drug-resistant forms. For its part, *T. brucei* can reside in the skin and other organs and remain undetected for a long time, even in the absence of detectable parasitemia. All this could contribute to refraction to drug treatment and, in turn, would imply the need for the development of new drugs and therapeutic alternatives for the treatment of these diseases [8,9,10,11]. Indeed, several therapeutic alternatives have been proposed for the treatment of these kinetoplastid diseases [12,13,14,15], including the use of antimicrobial peptides (AMPs) [16,17,18].

## 2. What Are Antimicrobial Peptides (AMPs)?

AMPs are a class of small peptides synthesized by pro- and eukaryotic organisms, used as a strategy for competition and defense during invasion by foreign organisms. They are encoded by specific genes and expressed constitutively or in response to specific environmental stimuli [19]. In some insects, AMPs are key for vector–microorganism interaction and are effective against both quiescent and actively proliferating pathogenic organisms [20,21,22].

These peptides are synthesized through three pathways, which include classical ribosomal synthesis, non-ribosomal synthesis, and proteolytic digestion of proteins. Ribosomally synthesized AMPs (RS-AMPs) are those encoded by genes and produced by ribosomal translation of specific mRNAs into the biologically active amino acids sequences. These AMPs are widely distributed in nature, produced by various organisms (such vertebrates, insects, plants, and bacteria) [23,24]. Among the RS-AMPs are the mammalian defensins and amphibian dermaceptins [23]. Non-ribosomally synthesized AMPs (NR-AMPs) are produced by enzymes known as non-ribosomal peptide synthases (NRPSs), which incorporate non-proteinogenic amino acids into the sequence and are found mainly in filamentous fungi and bacteria [24,25]. So far, hundreds of AMPs synthesized in a NRPS-dependent manner have been described, among which are gramicidin S and isopenicillin [26]. Other AMPs are produced via the proteolytic digestion pathway (peptides also known as cryptides) by proteases-mediated cleavage of precursor proteins or larger proteins with other functions, to yield matured bioactive factors [24,27]. During these processes, various fragmented peptides are also produced that can vary in their biological activity [27]. Buforin II is one of the most studied cryptid peptides [28].

**Figure 1:**
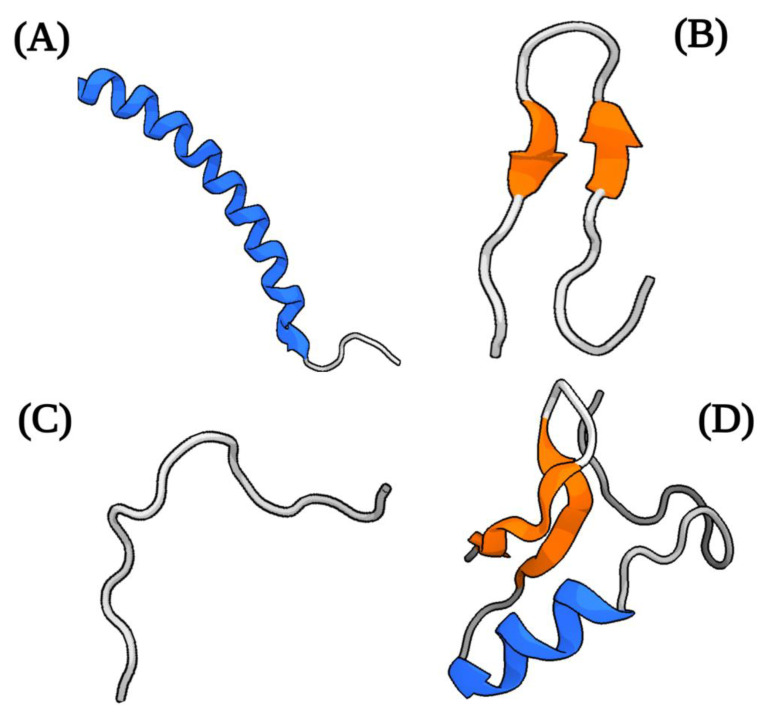
Structural classification common of naturally antimicrobial peptides (AMPs). Representative examples of common structural classes of AMPs. (**A**). α-Helical: structure of human cathelicidin LL-37 (PDB ID:2k60). (**B**). β-Sheet: polyphemusin I (PDB ID:1RKK). (**C**). Extended or loop: indolicidin (PDB ID:1G89). (**D**). Mixed (contain both α-helical and β-sheet elements): Defensin A (PDB ID: 1ICA). Created with BioRender.com (accessed on 20 February 2023).

Although natural AMPs are molecules with considerable diversity in their structural properties, origins, and mechanisms of action, they have certain characteristics in common. Generally, they are short molecules (≈10–100 amino acids) of a cationic nature at neutral pH (generally ranging from +2 to +11), which facilitates their interaction with charged cell membranes through electrostatic interaction [29,30]. Additionally, most AMPs have a considerable proportion of hydrophobic residues (close to 50%) and an amphipathic structure [30,31]. This latter property is responsible for their structural flexibility and solubility in aqueous environments. [30]. The overall positive net charge and amphipathicity are the two characteristics that contribute to the high affinity of AMPs for membranes [32]. Structurally, AMPs are commonly classified into four groups based on their secondary structure, which include linear α-helical peptides, β-sheet peptides (usually stabilized with one or more disulfide bonds), linear extension or loop (devoid of α- or β-elements) structure, and mixed (α-helical/β-sheet) peptides [29,30,32] (Figure 1). However, some peptides with cyclic structures and unusual complete topologies have also been documented [32]. Most studied among the groups of AMPs are the peptides with an α-helix structure [33].

**Figure 2:**
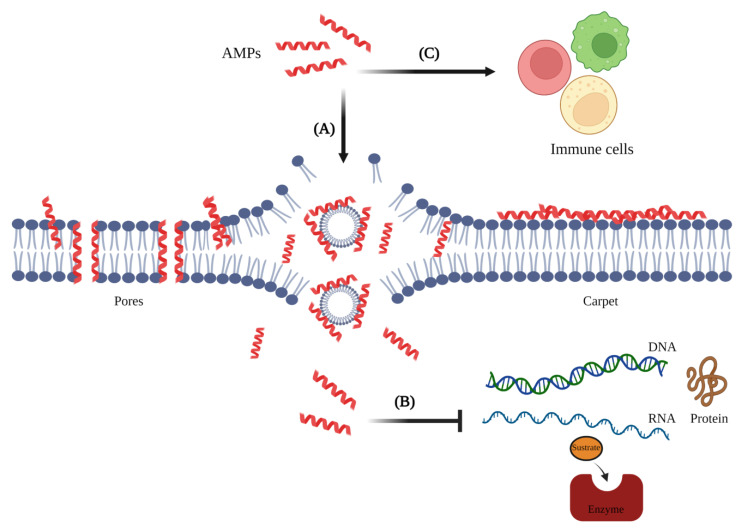
Mechanisms of action of antimicrobial peptides (AMPs). (**A**). Cell membrane damage. (**B**). Interaction with internal targets. (**C**). Modulation of the host innate immune response. Created with BioRender.com (accessed on 20 February 2023).

These peptides are characterized by having a broad-spectrum antimicrobial activity, which is attributed to mechanisms of action such as (A) cell membrane damage (promoting pore formation and development of a peptide “carpet” on the membrane surface), (B) interacting with internal targets (DNA or RNA, or interfering with protein synthesis or folding, or enzyme activity), and (C) modulation of the host innate immune responses [19,34,35,36] (Figure 2). Additionally, these AMPs have been shown to possess high specificity, limited toxicity, and a low probability of inducing resistance [35]. In this sense, AMPs have been proposed as an attractive therapeutic alternative for treatment of parasitic diseases [35,36,37].

The advantages of AMPs over other peptides provide the opportunity to develop them for therapeutic strategies. Compared to AMPs this may be more difficult to achieve with other peptides. For example, synthesis of synthetic peptides (SPep) requires, in some cases, very complex strategies and specialized and sophisticated equipment [38,39,40]. Additionally, the SPep may have heterogeneity within the chain associated with statistical copolymerization, which leads to an amino acid composition gradient. This makes identification of any structure–function correlations difficult [41]. Another advantage of AMPs is that their action does not depend on external factors (such as pH), whereas that of some Spep depends on microenvironmental conditions [38]. Also, AMPs have often low cytotoxicity, whereas for SPep, it has been documented that excessive positive charge density can lead to severe cell and tissue-based toxicity [42]. The use of AMPs with anticancer activity (“anticancer peptides” or ACPs) is considered a therapeutic strategy of great potential. Compared to specific-target drugs, these ACPs can act towards different intracellular targets in addition to presenting a mechanism of action at the membrane level, which would imply increasing the success of the therapy and a low propensity to resistance development. Additionally, few side effects are also a feature attributed to future ACPs-based therapies [31].

In this review, we will analyze AMPs as a possible therapeutic alternative for the treatment of trypanosomiasis, particularly emphasizing diseases caused by the parasites *T. brucei* and *T. cruzi*.

## 3. Why Is Trypanosomiasis Important?

Trypanosomiases form a set of diseases that affect millions of people and animals globally, especially in poor rural populations of the Americas, Asia, and sub-Saharan Africa [43,44,45]. These diseases are included in the group of Neglected Tropical Diseases (NTD), which attribute to a significant health, economic, and social impact in endemic regions [6,46,47].

Human African trypanosomiasis (HAT) or sleeping sickness is caused by two subspecies of *Trypanosoma brucei* that are pathogenic for humans and transmitted by tsetse flies, *Trypanosoma brucei gambiense* (*T. b. gambiense*) in western and central Africa, and *Trypanosoma brucei rhodesiense* (*T. b. rhodesiense*) in eastern Africa, with *T. b. gambiense* being responsible for more than 95% of all HAT cases [6,48]. In the early stages of HAT, symptoms are usually diverse and non-specific; however, in advanced stages of the disease, the severe symptoms are associated with central neurological impairment, and also involve weight loss, anemia, hepatosplenomegaly, arthralgia, and inflammatory processes [6,49,50,51]. This devastating disease threatens millions of people in sub-Saharan Africa, since it is estimated that 54 million live in areas with risk of infection [52]. It can even become the main cause of death in these communities, surpassing HIV/AIDS [6]. The economic losses to HAT exceed millions of dollars. Studies based on disability-adjusted-life years (DALYs) suggest that HAT causes approximately 1.6 million DALYs, which is why it is considered the second among all diseases in Africa for mortality and fourth for associated disabilities [53]. Also, it has been suggested that an elimination program could cost approximately US$ 1.2 billion [54].

American trypanosomiasis, also known as Chagas disease (CD), is transmitted by triatomine insects and caused by *T. cruzi* [55]. The clinical course of this disease is characterized by an acute phase, which may be asymptomatic or with nonspecific symptoms, followed by a chronic phase, in which there may also be a complete absence of signs and symptoms of the disease. However, in this chronic phase, 30–40% of patients develop multiorgan complications, mainly cardiomyopathy or megaviscera (megaesophagus, megacolon, or both), peripheral neuropathy, dermatological manifestations, and early death [55,56]. This anthropozoonosis has a globalized distribution; however, it is endemic to 21 countries in the Americas, affecting approximately 7–8 million people, most in rural areas, causing 50,000 deaths per year [57,58,59,60]. In the Americas alone, 30,000 new cases are reported each year, of which 8600 newborns are infected during pregnancy [61]. The annual global burden is US$ 627.5 million per year, mainly related to healthcare costs, of which 10% pertains to non-endemic countries [62,63]. In Latin America, the economic losses attributed to this disease are 752,000 working days because of premature deaths and US$ 1.2 billion in productivity [55,64].

## 4. Current Treatment of Trypanosomiases

Although standard therapies are available for treatment of trypanosomiases, these are mainly based on synthetic drugs mostly developed more than 40–50 years ago, several of them highly toxic, and their use depends on the stage of the disease and/or trypanosome species causing the infection [58,65].

Treatments for HAT involve five synthetic drugs, pentamidine, suramin sulfate, melarsoprol, nifurtimox/eflornithine combination (NECT), and fexinidazole. The mechanism of action of these drugs is mainly based on causing DNA damage and the inhibition of enzymes involved in various cellular processes of the parasite (DNA replication, glutathione metabolism, trypanothione biosynthesis, NADH/NAD^+^ balance maintenance, mitochondrial mRNA editing, and glycolysis) [65,66]. Most of these drugs are specific for treating infections caused by either *T. b*. *rhodesiense* or *T. b. gambiense*, except for suramin sulfate, used to treat infections caused by both parasites [65]. The efficacy of these drugs depends on the stage of the disease. Pentamidine and suramin are used during the initial stage of HAT (hemolymphatic), whereas melarsoprol, eflornithine, and NECT are used during the advanced stage of the disease, when parasites have migrated to the central nervous system. All these drugs require prolonged use, intravenous infusion, and are highly costly, often resulting in non-compliance and abandonment of treatment [65]. Also, the administration of these drugs generally has associated side effects that in some cases can be fatal and appear in the first days of treatment [65,67,68]. Another disadvantage is the development of resistance to these drugs that is mainly associated with the loss of function of the parasite’s transporters that mediate their internalization [65,69].

Recently, the US Food and Drug Administration (FDA) and the European Medicines Agency (EMA) authorized the marketing of fexinidazole (FNZ) [70,71], an oral nitroimidazole drug, for the treatment of both stage 1 and 2 *T. b. gambiense* HAT, however, it is little effective in patients with severe stage 2 HAT. Fexinidazole is a prodrug whose activity depends on electronic reductions, facilitated by a type-I NADH-specific nitroreductase (TbNTR1), which leads to the formation of reactive metabolites that can induce damage to the kinetoplast DNA (kDNA) and to the trypanosome nuclear genome and its proteins, as well as inhibition of DNA synthesis [65,72]. The decrease in the activity of this enzyme or the changes in its *tbntr1* gene, lead to resistance to fexinidazole and cross-resistance to other nitroheterocycles, including nifurtimox [73]. Although the side effects caused by FNZ are milder compared to those of the other drugs [74], its use is only recommended in patients who do not have other available treatment options. In infants, it is only recommended to be used at 6 years of age and older and weighing at least 20 kg [71,74,75], which means a limitation for the treatment of congenital HAT.

Regarding Chagas disease, currently only two drugs, benznidazole (BZN) and nifurtimox (NF) are licensed for the treatment of this disease. The mechanism of action of both drugs involves intracellular activation of a mitochondrial NADH-dependent type-I nitroreductase (TcNTR), which gives rise to intermediates (free radicals and/or electrophilic metabolites) that bind to intracellular macromolecules and inhibit several vital biological processes of the parasite (DNA synthesis, DNA and RNA metabolism, protein synthesis, and energy metabolism) [55,58]. The efficiency of NF and BZN depends on the stage of the disease. These drugs tend to be less effective in the chronic phase, where the cure figure hardly reaches 20–30% [76,77]. Although both compounds are administered orally in two or three doses, treatment is discontinued in 9–75% of patients due to severe side effects [58,78]. Additionally, the use of these drugs is not recommended during pregnancy and lactation, and in the case of NF, it is only approved for newborns over 2.5 kg [79,80], meaning a limitation for the prevention of vertical transmission of the parasite and timely treatment of congenital CD. The occurrence of resistance in strains, mediated by various mechanisms (e.g., loss/mutations/polymorphism of TcNTR) [81,82] are other limitations of the clinical use of BNZ and NF. Notably, these drugs cannot prevent or reverse the damage caused, especially in the heart, by inflammation in response to *T. cruzi* infection, even in conditions where a decrease in parasitic load has been observed [83,84].

FNZ and its derivatives have also been proposed as a therapeutic alternative in adults with chronic CD, since it has been shown that low FNZ doses can be safe and effective in treatment regimens of <10 days [85,86]. However, neutropenia, alterations in platelet counts and elevations in hepatic enzymes can be observed in patients, in a dose-dependent manner [86]. 

Most of the drugs currently available for the treatment of trypanosomiases have an ancient origin and high toxicity. Others, despite being specific and efficient for the early and advanced stages of infection, depend on an enzyme or membrane transporter of the parasite for their activation. The latter generally implies a probability of resistance development over time. For all these reasons, there is a need to develop or search compounds that will overcome these limitations observed in anti-trypanosome drugs available on the market. Although this search may seem very demanding, these “ideal drug candidates” could be found in various natural sources. This will be discussed in more depth in the next section.

## 5. Other Therapeutic Alternatives against Trypanosomiasis

All studies related to available drugs for the treatment of trypanosomiasis have highlighted the need to design new therapeutic strategies, either by optimization of existing drugs (in combination with other compounds) or by the formulation of new compounds. Salvage chemotherapy or repositioning of established pharmacotherapeutic agents, with known activity and side-effect profiles, have been considered as candidates for the treatment of trypanosomiases [58,65,66,77]. Several of these repositioned drugs are commonly used as dietary supplements and to treat other diseases (bacterial and fungal infections, hypertension, depression, osteoporosis) [77,87,88,89,90,91,92]. Many studies with such drugs are in the preclinical phase for trypanosomiasis (using methodologies based on in vitro or animal studies), clinical trials, and described in case reports. Also, synthetic, semi-synthetic [93,94,95,96,97], and natural compounds have been proposed as alternatives for the treatment of these trypanosomiases [98,99,100,101]. Among these natural compounds with anti-trypanosome activity, AMPs are included [16,102,103].

AMPs exert their antiparasitic effect against these parasites mainly through their association with, and subsequent rupture of the plasma membrane. However, they can also induce killing of the parasites through alteration of calcium homeostasis, and mitochondrial function, and induce activation of various cell-death pathways [102]. Additionally, anti-inflammatory properties have been attributed to these AMPs [104] with, in some cases, little or no toxicity against mammalian cells [105,106], and they exert their activity in very low concentrations [16]. All these attributes lead to postulating AMPs as attractive strategies for the treatment of trypanosomiases.

## 6. AMPs with Antiparasitic Activity

Several studies have shown the antiparasitic effect of some AMPs [35,107,108], including activity against parasites that cause important tropical diseases [109] (Figure 1). Many of these AMPs have been isolated from various vertebrate and invertebrate hosts of these parasites [107,110,111].

For apicomplexan parasites, most studies with AMPs have focused on *Plasmodium* spp. and *Toxoplasma gondii* [112]. These peptides have an inhibitory effect on the growth, life-cycle development, infectivity, and transmission of these parasites [113,114,115,116].

*Plasmodium* is the parasite on which most studies with AMPs have been carried out [112]. In this protist, many natural AMPs act primarily by disrupting the integrity of cell membranes [117,118,119,120,121,122]. However, some others can interfere with other important cellular processes of the parasite. In *Plasmodium berghei*, some fungal AMPs have an inhibitory effect on histone deacetylase (HDA), thus inducing histone hypermethylation and subsequent alteration of gene expression in the parasite [123]. Other AMPs derived from Gram-positive bacteria, such as epoxomicin and derivatives of the natural cyclic oligopeptide thiostrepton, have an inhibitory effect on protein synthesis and turnover, due to their binding to and inhibition of catalytic activity of proteasome β subunits (20S) [124,125]. Additionally, thiostrepton can inhibit mRNA translation in the apicoplast through its binding to the plasmodial organellar rRNA promoting structural alterations that prevent its function during protein synthesis [125,126]. Importantly, antimalarial activities have been attributed to some AMPs with semi-synthetic and synthetic origin. Synthetic AMPs inhibit the plasmodial cysteine protease falcipain and aspartic proteases plasmepsin I and plasmepsin II, involved in hemoglobin hydrolysis and hemozoin formation, thus interfering with parasite metabolism and growth [127,128,129]. Notably, some synthetic peptides have also shown an effect on some enzymes such as topoisomerase I, affecting the parasite’s DNA metabolism [130]. Several of these AMPs not only have antiplasmodial activity against different developmental stages of some *Plasmodium* species (*P. falciparum*, *P. berghei*, and *P. yoelii nigeriensis*) in vitro conditions [121,124,131], but are also effective at high parasitemia in an animal model [122].

In the case of *T. gondii*, the yeast killer toxin (KT) can induce apoptotic-like cell death [132]. Other AMPs such as apicidin, a fungal peptide, demonstrated in vitro activity against apicomplexan parasites, including *T. gondii*, through the inhibition of HDA [123]. This defensin can reduce the viability of the parasite and, consequently, host cell infection [133]. Because of their high specificity, some AMPs have inhibitory effects on apicomplexans parasites at pico- and nanomolar concentrations [112,120,124,127,134,135].

In helminths, studies have focused primarily on *Schistosoma* and *Brugia*. In these parasites, AMPs have effects on motility, development, egg deposition, and the integumentary surface [136,137,138,139,140]. In *Brugia pahangi*, synthetic cecropins A/B, AMPs from insect hemolymph, attenuate microfilariae mobility and larval development in adult female *Aedes aegypti* [136]. In *Schistosoma*, dermaseptin, a peptide isolated from frogs, can synergistically interact with other natural compounds and contribute to parasite killing and infection control. In combination with piplartine, an amide alkaloid of *Piper longum L*. (long piper), dermaseptin not only exerts activity against the *Schistosoma mansoni* (*S. mansoni*) stages (schistosomula and adult) and affects the reproductive fitness of adult worms, but also induces structural alterations of the tegument and extensive destruction of the tubercles [137,138]. Although the anthelmintic mechanism of AMPs has not been elucidated, it has been proposed that disruption of cell structure by pore formation by direct interaction with the lipid bilayer seems to be the most likely [136,138,141,142]. It should be noted that the integument is essential for the survival of the helminth parasites, since it is involved in nutrient absorption and in the interaction with the host [143,144,145,146]. In both *Brugia* and *Schistosoma*, divalent metal transporter 1 (DMT1) molecules are present in the integument and are essential for the absorption of iron, an essential ion for the development and reproduction of these parasites [144,146,147]. In this sense, directing AMPs against the tegument of these parasites could be a good anthelmintic strategy.

Unlike in apicomplexan parasites, the AMPs tested so far on helminths exert their antiparasitic action at micromolar concentrations [136,137,138]. Some AMPs with antimicrobial properties have been discovered in helminths [142,148,149]. In *S. mansoni*, an AMP called schistocins has been obtained from the protein SmKI-1, a key protein for the survival of this nematode, which has activity against *Schistosoma* itself [142]. Likewise, putative neuropeptides derived from this parasite alter the behavior of the cercariae stage, therefore their use has been proposed as strategy for the control of the infection [140].

In trypanosomes such as *Trypanosoma evansi* and *Trypanosoma equiperdum*, causing surra and dourine in animals, some AMPs have been shown to exert an trypanocidal effect; hence, they have been proposed for use in new treatment strategies of trypanosomiasis in animals [150,151]. Furthermore, AMPs isolated from triatomine hemolymph have been shown to have trypanolytic activity against different strains of *Trypanosoma rangeli*, an infectious but non-pathogenic human parasite [152]. In these trypanosomes, AMPs exert their action through different mechanisms, including plasma membrane permeabilization, mitochondrial alteration, and parasite lysis [150,151,152].

In the following sections, we will analyze AMPs as antiparasitic agents against the trypanosomes *T. brucei* and *T. cruzi*, etiologic agents of trypanosomiases in humans.

## 7. Antimicrobial Peptides against Kinetoplastids Causing Neglected Tropical Diseases

Various AMPs with trypanocidal activity against *T. brucei* and. *T. cruzi* have been identified, some of which have been found in host organisms for these parasites (Supplementary Table S1).

### 7.1. AMPs against T. brucei

**Figure 3:**
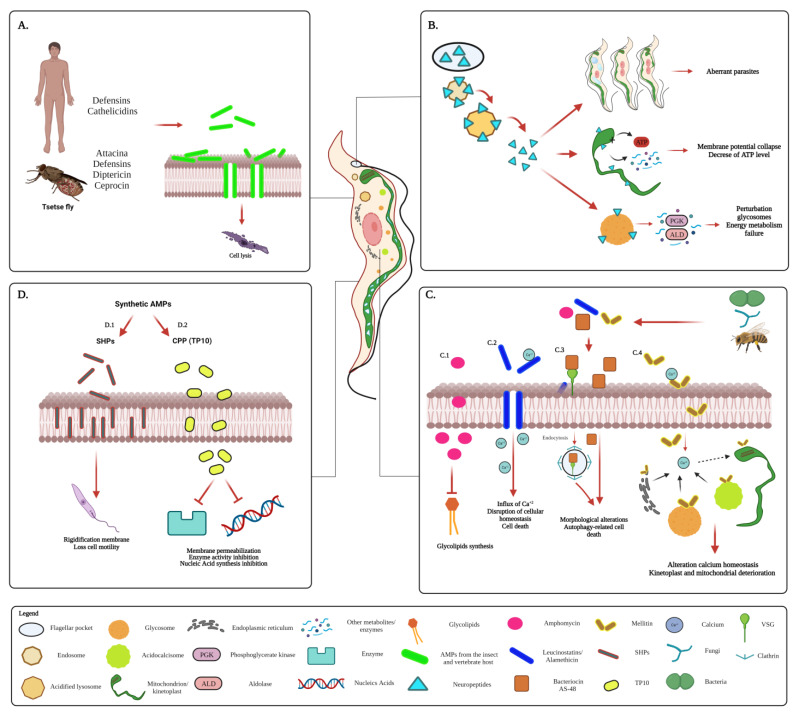
Mechanisms of action of antimicrobial peptides (AMPs) against *T. brucei.* (**A**). AMPs derived from the vertebrate host and insect vector. Peptides isolated from both vectors and hosts exert their trypanocidal effect through membrane perturbation and induction of cell lysis. (**B**). Mechanism of action of neuropeptides (NPs). The killing of trypanosomes by NPs requires the NPs to be endocytosed through the flagellar pocket and transported from the endosomes to the acidified lysosome, where they break the lysosomal bilayer membrane and accumulate in the cytoplasm. Once in the cytoplasm, their interference in various cellular processes contributes to morphological alterations and disturbance of organelles (glycosomes and mitochondrion), which ultimately lead to depletion of ATP and failure of the energy metabolism. (**C**). AMPs isolated from natural sources (bacteria, fungi, and insects). (C.1,C.2). AMPs derived from fungi. The lipopeptide amphomycin, inhibits the biosynthesis of the glycolipid precursor of glycosylphosphatidylinositol (GPI) by which the variant surface glycoproteins (VSGs) are anchored to the plasma membrane of these parasites (C.1). Leucinostatins (A and B) and alamethicin act as ionophores and pore formers in the membranes, causing alteration of cellular homeostasis, ultimately leading to the death of the parasite. (C.3). AMPs isolated from bacteria. Bacteriocin AS-48 targets intracellular compartments without plasma membrane permeabilization. AS-48 may interact at the surface with VSGs and then promotes its internalization through a clathrin-mediated endocytic process. In the cytoplasm, it induces structural alterations and autophagy-like cell death. (C.4). AMPs derived from bee venom. Melittin induces an increased influx of Ca^2+^ through the plasma membrane or increased release from acidocalcisomes. Excess Ca^2+^ accumulated intracellularly is stored in the mitochondrion, causing a reduced mitochondrial membrane potential, disorganization of kinetoplast DNA, autophagy, and cell death. (**D**). Synthetic AMPs. (D.1). SHPs intercalate and insert deeply into the plasma membrane, resulting in changes in the distribution of membrane components, increased membrane stiffness, loss of cell motility, and cell death. (D.2). For their part, the CPPs cross the membrane, accumulate in the cytoplasm and interfere with various cellular processes (such as inhibition of metabolic enzymes and RNA/DNA synthesis). Created with BioRender.com (accessed on 23 November 2022).

Many of the AMPs that are active against *T. brucei* are produced by a wide variety of organisms, including mammals and the insect vector [16,153]. These can carry out their action extracellularly, by plasma membrane disturbance, or intracellularly, by altering the function of some intracellular compartments [16,16,153,154] (Figure 3).

Among the AMPs from insects, some peptides found in species of the tsetse fly *Glossina* are highlighted in Figure 3A. These AMPs include attacina, defensins, diptericin, and cecropin, involved not only in the antimicrobial response against African trypanosomes but also in immunomodulatory functions [16,153,155]. All these AMPs, derived from hemolymph, fat body, and proventriculus, are associated with the response to infection by trypanosomes [156]. These peptides have an effect in the micromolar concentration range against the mammalian bloodstream form (BSF) and insect-stage procyclic form (PCF) of *T. brucei* [16,153,154], through permeabilization of the parasite’s plasma membrane, via interaction and formation of pores [153,156,157] (Figure 3A). In addition, the AMP stomoxyn from another fly species, *Stomoxys calcitrans*, a not-cyclical vector of trypanosomes and sympatric with tsetse flies, exhibits trypanolytic activity to BSF *T. b. rhodesiense* [158].

Vertebrate host-derived peptides are the most studied AMPs with anti-trypanosome activity [16,155,159]. The defensins and the cathelicidins are mammalian AMPs that have been shown to exert a trypanolytic effect by membrane permeabilization and disruption of internal structures [16,159] (Figure 3A). Under in vitro conditions, human β-defensins, exhibits very weak killing of the PCF and BSF forms of *T. brucei*, only obtaining a reduction in survival (18–33%) when the parasites were incubated with this peptide [159]. Other defensins of mammal such as cryptdin-4, a murine α-defensin, also exhibits similar weak killing of the PCF of *T. brucei*, when the parasites were incubated with this peptide [159]. However, some cathelicidins, such as LL-37, were more effective in killing both the PCF and BSF of the parasite because 100% reduction in survival of parasites was found when incubated with these AMPs [155]. Similarly, other some cathelicidins, such as SMAP-29 and protegrin-1, were effective in killing both the PCF and BSF of the parasite because 39%–95% reduction in survival of parasites was found when incubated with these AMPs. Additionally, the administration of these cathelicidins to *T. brucei*–infected mice decreased parasitemia and prolonged survival of the animals [159]. Both peptides, defensins and cathelicidins, exert their trypanolytic activity in the micromolar concentration range [16,155,159,160]. The cationic nature of these AMPs may allow them to more easily interact with the negatively charged cell surface of trypanosomatids, mainly due to the presence of sialic acids associated with glycoproteins, glycolipids, and of phosphate groups [161]. Alternatively, the susceptibility of *T. brucei* membranes to AMPs could also be related to the abundance of glycosylphosphatidylinositol (GPI) protein anchors on their surface [159].

Other cathelicidins from sheep (OaBAC-5-mini) and bovine (BMAP-27, indolicidin, BAC-CN) have a trypanolytic effect on both the PCF and BSF of *T. brucei* [155,160].

Other studies have evaluated the trypanocidal ability of neuropeptides (NPs) [154], soluble mediators produced by the human neuroendocrine and immune system, which participate in functions related to regulating physiological homeostasis, neuroprotection, immunomodulation, and antimicrobial properties [162,163,164]. These NPs exert their parasitic effect through a mechanism different from that described for other AMPs [159]. Some neuropeptides, such as vasoactive intestinal peptide (VIP), alpha-melanocyte-stimulating hormone (α-MSH), urocortin (UCN), adrenomedullin (AM), ghrelin (GHR), and corticotropin-releasing hormone (CRH), can kill the BSF of the animal- but not human-infective subspecies *T. brucei brucei* (*T. b. brucei*) by targeting intracellular compartments and inducing autophagy-like cell death [154] (Figure 3B). These NPs are endocytosed through the flagellar pocket and enter the normal trafficking pathway of the parasite. Subsequently, they disrupt lysosome integrity and accumulate intracellularly, finally causing disruption of intracellular compartments and killing the trypanosome. Some NPs induce morphological alterations such as cell size, formation of vacuolar-like structures, detachment of the flagellum, and consequent reduced motility. Additionally, they can induce a block in cytokinesis, leading to the presence of aberrant parasites with two mitochondria or kinetoplasts (Figure 3B). Also, some NPs such as VIP alter intracellular trafficking, reduce the mitochondrial membrane potential and decrease the ATP level. In BSF parasites, which are dependent on glycosomal metabolism for energy, VIP causes disturbance of glycosomes with partial relocalization of some glycolytic enzymes, phosphoglycerate kinase (PGK), and aldolase (ALD), to the cytosol. All these events together lead to energy metabolism failure that initiates the autophagy-like cell death. Finally, rupture of the plasma membrane and cell disintegration occurs (Figure 3B) [154,165]. Although these NPs induce the death of trypanosomes through a cascade of events, it should be noted that the mechanism by which they exert their effect depends on their cationic nature, which allows them to recognize and interact with the anionic residues exposed on the plasma membrane. All these NPs have inhibitory effects in the micromolar range [154].

AMPs with activity against *T. brucei* have also been isolated from other natural sources (Figure 3C) [166,167,168,169]. The antibiotic peptides, amphomycin, leucinostatins, and alamethicin, isolated from fungal species have a trypanocidal effect against *T. brucei* species [166,167,170] (Figure 3C.1). The lipopeptide amphomycin, isolated from *Streptomyces canus* was active against BSFs of both subspecies *T. b. gambiense* and *T. b. rhodesiense*, leading to a definitive cure of the infection in mice when it was administered on four successive days [166]. This antibiotic inhibits the biosynthesis of the glycolipid precursor of GPI by which the variant surface glycoproteins (VSGs) are anchored in the membrane of these parasites [166,170]. In this sense, it would be valid to think that this peptide could influence the antigenic variation of the parasite, a key process for immune evasion [171]. For their part, the antibiotic peptides, leucinostatins (A and B) and alamethicin, isolated from *Paecilomyces* spp., exhibit also potent anti-trypanosomal activity against BSFs of *T. b. brucei* and *T. b. rhodesiense*, with even up to 200 times higher activity than suramin and with little cytotoxicity in human cell lines. These peptides act as ionophores and pore formers in the membranes, causing disruption of cellular homeostasis, ultimately leading to the death of the parasite [167,172] (Figure 3C.2). Specifically, by acting as a divalent ionophores, leucinostatins A and alamethicin mediate Ca^2+^ entry into the cells [173,174]. The increased influx of Ca^2+^ then can induce alterations in the different cellular signaling pathways where this ion acts as a second messenger, which are essential in the physiology of *T. brucei* [167,175]. Additionally, the internal environment of some intracellular compartments where Ca^2+^ is stored, such as acidocalcisomes, mitochondrion, and endoplasmic reticulum [175], would be perturbed. It could be hypothesized that all this would cause prolonged elevated levels of intracellular Ca^2+^ that lead to cell death.

For their part, AMPs isolated from bacteria have also been tested against the different *T. brucei* subspecies [168,169]. Bacteriocin AS-48 has the ability to kill BSFs of *T. b. gambiense, T. b. rhodesiense*, and *T. b. brucei*, through targeting intracellular compartments without plasma membrane permeabilization. AS-48 may interact with VSGs on the surface and promote clathrin-mediated endocytosis of VSG-bound AS-48. In the cytoplasm, AS-48 induces structural alterations, such as the formation of multilamellar vesicles, myelin-like structures, alteration of the nuclear envelope, and autophagy-like cell death. This AMP has an anti-trypanosomal activity at concentrations in the low nanomolar range and is innocuous to mammalian cells [169] (Figure 3C.3). Some of these peptides isolated from entomopathogenic bacteria have activity against *T. b. rhodesiense*; however, their possible mechanism of action is still unknown [168].

Amphiphilic peptides such as melittin, the main component of apitoxin (the bee venom), can induce alteration of Ca^2+^ homeostasis in protistan pathogens, including *T. b. brucei* [176,177]. This peptide promotes an increased influx of Ca^2+^ through the plasma membrane or release from acidocalcisomes. The excess Ca^2+^ accumulated intracellularly is then stored in the mitochondrion, reducing the mitochondrial membrane potential, disorganizing kinetoplast DNA, and promoting autophagy and cell death [176,178] (Figure 1C.4). It is noteworthy that in kinetoplastids such as *Leishmania*, the presence of Ca^2+^ in other structures such as glycosomes has been reported [179]. Whether this is true accumulation remains to be confirmed. Ca^2+^ storage in glycosomes has not been documented for trypanosomes. Melittin could likely disturb Ca^2+^ distribution in these parasites and consequently affect their metabolism (Figure 3C.4). Therefore, melittin has been proposed as a therapeutic agent against these parasites.

The trypanocidal effect of some synthetic peptides has also been evaluated [180,181,182]. Specific small hydrophobic peptides (SHPs) trypanolytic for the BSF of *T. b. brucei* have been reported. The toxic activity of such peptides is conferred by their hydrophobicity and charge distribution, with their ability to intercalate and insert deeply in the membrane, which results in changes in the distribution of membrane components and subsequently, increased rigidity of the plasma membrane, loss of cell motility, and cell death [181,182]. Importantly, BSFs of *T. vivax* and *T. congolense*, the *Trypanosoma* species responsible for most cases of trypanosomiasis in domestic animals, are susceptible to killing by some peptides, such as SHP-1, at concentrations similar to those for BSF *T. b. brucei* (Figure 3D). This suggests that the susceptibility to these SHPs is a characteristic common of both human and veterinary pathogenic African trypanosomes [182] (Figure 3D.1). Other peptides such as cell-penetrating peptides (CPPs), specifically TP10, a derivative of bovine BMAP-27, can accumulate within the cytoplasm to carry out their antiparasitic activity against BSFs of *T. b. brucei* [180] (Figure 3D.2). In the intracellular environment, TP10 interferes with cellular processes such as enzymatic activities and nucleic acid synthesis [180,183]. Both synthetic peptides groups, SHP and CPPs, exert their anti-trypanosome effect in the micromolar concentration range [180,182].

### 7.2. AMPs against T. cruzi

**Figure 4:**
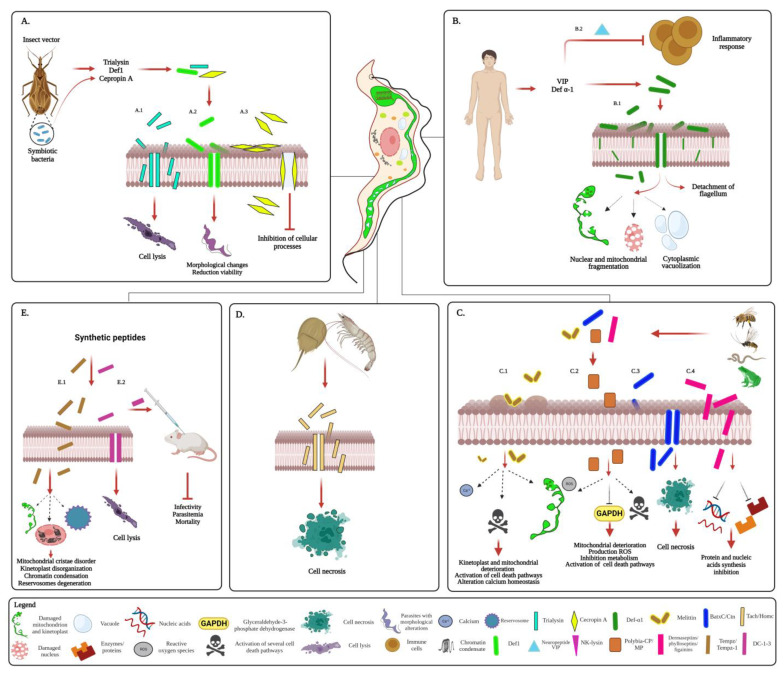
Mechanisms of action of antimicrobial peptides (AMPs) against *T. cruzi*. (**A**). AMPs derived from the insect vector. These AMPs carry out their activities by disturbing the plasma membrane and forming pores in it. (A.1). Trialysin induces cell lysis. (A.2). Def1.3 promotes morphological alterations, reduced viability, and inhibits growth of the parasites. (A.3). Cecropin A perforates the plasma membrane, causing cell lysis. (**B**). AMPs derived from the human host. (B.1). Def-α-1 exerts its trypanocidal effect through membrane pore formation, cytoplasmic vacuolization, and the induction of nuclear and mitochondrial DNA fragmentation, and detachment and release of the flagellum, leading to parasite destruction. Preincubation of parasites with this peptide inhibits their infective ability and causes reduction of the parasitemia. (B.2). The neuropeptide VIP modulates the inflammatory response to *T. cruzi*, reducing cardiac damage. (**C**). AMPs derived from other natural sources (insects, reptiles, and amphibians). (C.1) Melittin induces structural changes (including disruption of the plasma membrane, structural changes in the mitochondrion, kinetoplast disorganization, and structural alterations of the flagellum), alteration of Ca^2+^ homeostasis, and activation of different cell death pathways in the parasite. (C.2). Polybia-CP and MP carry out their trypanocidal effect through the promotion of ROS, mitochondrial dysfunction and apoptosis-like cell death. Additionally, MP can inhibit the glycolytic enzyme GAPDH. (C.3). BatxC and Ctn induce the formation of pores in the plasma membrane, promoting the formation of ROS, loss of the mitochondrial membrane potential, and cell death by necrosis. (C.4). Peptides dermaseptins 1/4 and phylloseptins 7/8 have trypanocidal activity through disruption of the plasma membrane and effect on several intracellular targets (such as protein and nucleic acids synthesis). (**D**). AMPs derived from aquatic organisms. Peptides isolated from marine organisms (Tach and fragments from hemocyanin) have anti-*T. cruzi* activity by causing structural alterations in the plasma membrane and the formation of pores, and subsequent activation of cell death by necrosis. (**E**). Synthetic AMPs. (E.1). Tempz and Tempz-1 have toxicity against *T. cruzi* through cytoplasmic alterations in the parasite. These alterations are related to chromatin condensation, mitochondrial cristae disorder, kinetoplast disorganization, and an increased number and degeneration of reservosomes. (E.2). For their part, DC1-3 lytic peptides carry out their trypanocidal activity by perforation of the plasma membrane and subsequent cell lysis. Some of these peptides decrease the infectivity of the parasite, as well as the parasitemia and mortality of mice infected with *T. cruzi*. Created with BioRender.com.

The antiparasitic activity of AMPs has also been evaluated on *T. cruzi*, using some peptides obtained from a variety of natural sources and others synthetically prepared [17,22,103,106,178,184,185] (Figure 4).

AMPs obtained from various triatomine species, including some *T. cruzi*-transmitting vectors, have been shown to possess activity against this parasite. Some of these are involved in the defense response against infection and control of parasitemia in the vector [20,22,186,187] (Figure 4A). From the saliva of *Triatoma infestans*, the trialysin peptide was isolated, which has a cytotoxic activity against the infective (metacyclic trypomastigote (Tryp) and replicative (epimastigote (Epi)) insect stage of the *T. cruzi* Y strain (a BZN-resistant strain), through the formation of pores in the membrane [20] (Figure 4A.1). In *Triatoma (Meccus) pallidipennis*, defensins 1.3 (Def1.3) have trypanocidal activity against the parasitic kinetoplastids, including the *T. cruzi* TBAR/MX/0000/Querétaro strain (Qro)*,* inducing morphological alterations, reduced viability and inhibited growth [22] (Figure 4A.2). It should be noted that the Qro isolate is a highly virulent parasite that under experimental conditions causes 100% mortality in mice. This mortality is attributable to the exacerbated inflammatory process induced and the damage caused by it in cardiac tissue [188].

Trypanolytic factors with activity against *T. cruzi* from different discrete taxonomic units (DTU): TcII, TcV, TcVI Tcba, and Tcmarinkellei have also been identified in the hemolymph of the triatomines *Rhodnius prolixus* and *Rhodnius robustus*. The lytic activity of these factors is independent of the developmental stage and sex of the vector, and the blood source [152]. Although the chemical structures and mechanism of action of these lytic factors are unknown, it has been shown that these factors or their precursors were proteins or AMPs [189].

Notably, a recombinant *Rhodococcus rhodnii* has been engineered that expresses cecropin A. *R. rhodnii* is an obligate symbiotic bacterium of some *T. cruzi* vectors where it is required in the hindgut lumen for the insect’s survival. Cecropin A is an AMP which has activity against several strains of *T. cruzi*, including strains Y and DM28, through membrane perforation and subsequent lysis, due to loss of osmotic equilibrium of the cell [186,190]. When this peptide is expressed by *R. rhodnii*, in the intestine of the triatomine vector, it induces the lysis of Epi and metacyclic trypomastigotes in the hindgut, and consequently clearance of the infection in the vector [186,190] (4A.3). This paratransgenic strategy could represent a novel alternative for control of vectorial transmission of *T. cruzi*, especially relevant because of the increasing resistance of vectors to insecticides [191,192,193]. All these AMPs isolated from insect vectors and their symbionts such as *R. rhodnii* exert their trypanocidal effect at micromolar concentrations; however, some of them, such as trialysin, are cytotoxic to host cells [20,22,186,187,190].

Although there are few reports about human AMPs with trypanolytic effect, some peptides such as defensin (Def) have an antiparasitic effect against *T. cruzi* [194,195]. In vitro studies evidenced that the defensin α-1 (Def- α-1) has a trypanocidal activity against Tryp and Epi forms of *T. cruzi* clone MMC 20A, through membrane pore formation, cytoplasmic vacuolization, and the induction of nuclear and mitochondrial DNA fragmentation, leading to parasite destruction. Additionally, preincubation of Tryp with peptide (Def- α-1), inhibited the infective ability of the parasites exposed to epithelial cells, consequently reducing the infection of the host cells [195]. Alternatively, Def- α-1 reduces infection because of its binding to the flagellar membrane and axoneme, leading to breakage of the flagellar membrane, and detachment and release of the flagellum from the parasite [196] (Figure 4B.1). Notably, Def- α-1 are overexpressed in human cells in response to early *T. cruzi* infection as a mechanism to modulate parasite load, by induction of apoptotic death of trypomastigotes, and an effective host innate immune response to control *T. cruzi* infection [197]. It is important to highlight that defensins are key peptides in the innate immune responses due to their antimicrobial, chemotactic, and regulatory activities. This may raise the suggestion that the use of molecules mimicking some critical peptides of the innate immune response early during a *T. cruzi* infection could be a therapeutic strategy for the treatment of Chagas disease.

Interestingly, in studies evaluating the effect of VIP on systemic and cardiac immune responses during experimental acute infection in mice, it was shown that this NP can reduce the inflammatory response to the *T. cruzi* VL-10 strain, limiting cardiac damage [104] (Figure 4B.2). VIP is a potent anti-inflammatory factor, both in innate and adaptive immunity, which carries out its biological functions through the binding of G protein-coupled receptors, VPAC1 and VPAC2, and subsequent activation of the cAMP/PKA pathway, involved in the regulation of the inflammatory response by immune cells [198,199]. Low levels of this NP are associated with Chagas disease cardiomyopathy [199]. This immunomodulatory capacity and possible trypanocidal activity of VIP are characteristics that could influence its use in the treatment of CD, especially in advanced stages where a chronic self-destructive immune response is observed [200].

Other studies have reported the trypanocidal effect of AMPs produced by various other organisms [103,106,178,185,201]. Some AMPs have been isolated from the venom extract of insects and reptiles [201,202,203,204] (Figure 4C). Similar to what was observed for *T. brucei*, melittin is lethal for different developmental stages of the *T. cruzi* CL Brener clone. Exposure to these AMPs induces structural changes, including disruption of the plasma membrane, structural changes in the mitochondrion, kinetoplast disorganization, structural alterations of the flagellum, and activation of different cell death pathways in the parasite (Figure 4C.1). The activation of these pathways depends on the developmental stage of the parasite. Although necrotic cell death was induced in each of the different forms of *T. cruzi,* autophagy- and apoptosis-like cell death appeared to be the main death mechanism in epimastigotes and trypomastigotes, respectively. This peptide melittin exerts its perturbation initially through vesicle formation and disruption of the plasma membrane, to later activate the different cell death signaling pathways [202] (Figure 4C.1). Although the mechanism by which melittin activates several cell death pathways in *T. cruzi* is unknown, it is probable that the peptide stimulates proteins involved in the respective routes to cell death. Melittin is known to have the ability to stimulate G-proteins [205], which are implicated in numerous cellular signaling processes, including apoptosis and regulation of autophagy [206,207]. In the *T. cruzi* Y strain, melittin has been shown to cause alterations in Ca^2+^ homeostasis, mediated by the activation of phospholipase A2 [177], while in the *T. cruzi* macrophage tropic Tehuantepe strain it exerts its effect mainly through the inhibition of parasite motility and infectivity [194]. In all these strains, melittin has an antiparasitic effect between nano- and micromolar concentrations [177,194,202] and can act synergistically or additively with other AMPs to eradicate *T. cruzi* in vitro conditions [186].

For its part, polybia-CP and mastoparan (MP), AMPs isolated from the venom of the wasp *Polybia paulista*, have an effect against all developmental stages of the *T. cruzi* Y strain, through the induction of reactive oxygen species (ROS), mitochondrial dysfunction, and apoptosis-like cell death [201,208] (Figure 4C.2). Furthermore, in the case of MP, the peptide can interfere in carbon and energy metabolism by binding and inhibition of *T. cruzi*’s glyceraldehyde-3-phosphate dehydrogenase (TcGAPDH), a glycosomal enzyme of the glycolytic pathway and essential for parasite survival [208]. One of the most important aspects is that this AMP, at low concentrations, not only has an inhibitory effect on the proliferation of the intracellular amastigote form, responsible for maintaining *T. cruzi* infections and the development of not-proliferating amastigotes [9,11], but also on the process of invasion of the host cell [209,210].

Other AMPs such as batroxycidin (BatxC) and crotalicidin (Ctn), isolated from *Bothrops atrox and* *Crotalus durissus terrificus*’s venom gland, induced death of all developmental stages of the *T. cruzi* Y strain through the formation of pores in the plasma membrane, promoting the production of ROS, loss of the mitochondrial membrane potential, and finally, cell death by necrosis [203,204]. Remarkably, some of these AMPs from venom extract, polybia-CP, BatxC, and Ctn, induce *T. cruzi* cell death with high selectivity (>100) when compared with some drugs such as BZN, which has a selectivity index (SI) of only 2.18 in the Y-strain that has acquired resistance against this drug [201,203,204]. Everything described so far points to AMPs from venoms as potential candidates for the design of anti-Chagas drugs.

AMPs obtained from some amphibians have been shown to have an anti-protistan effect (Figure 4C.4). These peptides obtained from skin secretion from different species of the frog *Phyllomedusa*, such as dermaseptins1/4 and phylloseptins 7/8, have trypanocidal activity against bloodstream trypomastigotes of the *T. cruzi* Y strain [105,106]. Also, figainin 1 and 2 cationic peptides, isolated from cutaneous secretions by the frog *Boana raniceps*, exhibited anti-epimastigote activity [211,212]. All these amphibian AMPs have an anti-*T cruzi* effect in the micromolar concentration range and their mechanism of action is through disruption of the cell membrane and effect on intracellular targets (such as synthesis of proteins, DNA and RNA) [106,211,212,213] (Figure 4C.4). Importantly, some of these AMPs, such as dermaseptin, have no toxicity to mammalian cells, which would suggest that they could serve as a mold for anti-*T. cruzi* drug design [105,106].

Anti-*T cruzi* activity of AMPs isolated from aquatic organisms has also been reported [103,106,185] (Figure 4D). Tachyplesin (Tach), isolated from the crab *Tachypleus tridentatus*, killed completely Tryp of the *T. cruzi* Y strain at micromolar concentrations with scant cytotoxic effect against mammalian cell lines. Also, Tach has leishmanicidal activities [185]. Although the exact mechanism of action in *T. cruzi* is unknown, it has been documented that this peptide forms transient pores in membranes and translocates across the membranes upon pore disintegration [214]. It was also shown that fragments from the hemocyanin of *Penaeus monodon* have activity against the Epi and Tryp of *T. cruzi*, through structural alterations in the plasma membrane, by the formation of pores and subsequent activation of cell death by necrosis [103] (Figure 4D). Although these marine AMPs exert their effect at micromolar concentrations, they have low selectivity [103,106,185], which would imply the need for some modifications to reduce their effect on host cells.

The design of synthetic AMPs has been considered a promising therapeutic strategy for Chagas disease [17,184] (Figure 4E). Temporizin (Tempz) and temporizin-1 (Tempz-1) are artificial hybrid peptides containing the N-terminal region of temporin A, a member of a larger temporin family found in skin secretion from frogs of the Ranidae family, and a C-terminus consisting of alternating leucine and lysine residues. These peptides have toxicity against *T. cruzi* because they promote cytoplasmic alterations in the parasite, associated with chromatin condensation, mitochondrial cristae disorder, kinetoplast disorganization, and an increase in the number and degeneration of reservosomes [17]. Other cecropin-like lytic (DC1-3) peptides, synthesized with virtually no sequence similarity with the natural compound (cecropin B), can kill all developmental stages of the *T. cruzi* Y strain under in vitro conditions. Some of these DC peptides influence the infectivity of the parasite, as well as the parasitemia and mortality of *T. cruzi*-infected mice [184] (Figure 4E). It should be noted that both Tempz/Tempz-1 and DC1-3 peptides exert their antiparasitic activity against *T. cruzi* at micromolar concentrations while exhibiting very low toxicity to mammalian cells [17,184].

## 8. Conclusions

AMPs are small peptides that have been shown to possess activity against different strains of *T. cruzi* and *T. brucei*, exerting their specific effect through different mechanisms such as rupture of the plasma membrane, alteration of calcium homeostasis, inhibition of some metabolic pathways, disturbance of organelles, and activation of various cell death pathways. Many of them have been shown to carry out their activity against the different developmental stages of trypanosomes. Some of them may also have activity against other kinetoplastids such as *Leishmania* spp. Additionally, most of them have no or only low toxicity towards mammalian cells and little anti-inflammatory effects. All these attributes render AMPs promising tools for the design of novel trypanocidal agents. It seems appropriate to consider them as candidates for further investigation and possible application as new therapeutic agents for trypanosomiasis and other diseases caused by kinetoplastids, either as an alternative or administered in complementary strategy to conventional treatments. Likewise, they could be used as a template for the design of analogous molecules with greater trypanocidal potency and/or reduced cytotoxicity on the host. 

## Data Availability

Not applicable.

## Supplementary Materials

The following supporting information can be downloaded at: https://www.mdpi.com/article/10.3390/biom13040599/s1, Table S1: Antimicrobial peptides with trypanocidal activity against *T. brucei* and *T. cruzi.* [215,216,217,218,219,220,221,222,223,224,225,226,227,228,229,230,231,232,233,234,235,236,237,238].
