# Supplementary material for: Antimicrobial Peptides (AMPs): Potential Therapeutic Strategy against Trypanosomiases?
Source: Biomolecules. 2023 Mar 26;13(4):599. doi: 10.3390/biom13040599 (PMC10135997; doi:10.3390/biom13040599)
Supplement: Supplementary file 1 [file biomolecules-13-00599-s001.zip › biomolecules-2203551-supplementary.pdf]

biomolecules MDPI

Table S1. Antimicrobial peptides with trypanocidal activity against *T. brucei* and *T. cruzi*

Target organism	Peptide	Origin	Structure	Sequence	Mechanism of action	Reference
T. brucei	Attacin	Tsetse fly *Glossina* (Fat body, proventriculus and haemolymph)	β-sheet	NANVVGGVFAAGNTDGGPATRGAFLAANKDGHGL SLQHSKTDNFGSSLTSSAHAHLFNDKTHKLDANAFH SRTHLDNGFKFDRVGGGLRYDHVTGHGASLTASRIP QLDMNTLGLTGKANLWSSPNRATTLDLTGGVSKHF GGPFDGQTNKQIGLGLNSRF	Disruption of the plasma membrane	[16,156,215,216]
	Defensin	Tsetse fly *Glossina* (Fat body, proventriculus and haemolymph)	Mixed (α-helix/β-sheet)	VTGELKRDKRVTCNIGEWVCVAHCNSKSKKSGYCSR GVCYCTN	Disruption of the plasma membrane	[156,157,216]
	Diptericin	Tsetse fly *Glossina* (Midgut, fat body and proventriculus)	β-sheet	PQSPPAQIKDPKIYASGGGSPKDGYNVNVDVRKNVWV SQNGRHSIDATGGYSQHLGGPYGNSRPDFRGGASYTYRF	Disruption of the plasma membrane	[16,157,216]
	Cecropin	Tsetse fly *Glossina* (haemolymph)	α-helix	GWLKKIGKKIERVGQNTRDATVKGLEVAQQAANVAAT-VRG	Disruption of the plasma membrane	[16,156,216–218]
	Stomoxyn	*Stomoxys calcitrans* midgut	α-helix	ARGFRKHFNKLVKKVKHTISETAHVAKDTAVIAG-SGAAVVAATG	Disruption of the plasma membrane	[16,158,216]
	β-defensin-1	Human epithelium	Mixed (α-helix/ β-sheet)	GLGHRSDHYNCVSSGGQCLYSACPIFTKIQGTCYR-GKAKCCK	Disruption of the plasma membrane	[216,219,220]
	β-defensin-2	Human epithelium, keratinocytes		GIGDPVTCLKSGACHPVFCPRRYKQIGTCGLPGTKCCKKP		[216,220,221]
	Cryptdin-4 (α-defensin)	Mouse paneth cells	β-sheet	LRGLLCYRKGHCKRGEEVRGTCGIRFLYCCPRR	Disruption of the plasma membrane	[216,218]

	LL-37	Human neutrophils	α-helix	LLGDFFRKSKEKKIVQRIKDFLRNLVPRTES	Disruption of the plasma membrane	[16,216,222–224]
	SMAP-29	Ovine myeloids	α-helix	RGLRRLGRKIAHGVKKYGPTVLRIIRIAG	Disruption of the plasma membrane	[16,216,225,226]
	OaBAC-5-mini	Ovine myeloids	α-helix	RFRPPIRRPPIRPPFRPPFRPPVR	Disruption of the plasma membrane	[16,155,225]
	BMAP-27	Bovine neutrophils	α-helix	GRFKRFRKKFKKLFKKLSPVIPLLHLG	Disruption of the plasma membrane	[160,216,227]
	Indolicidin	Bovine neutrophils	Loop	ILPWKWPWWPWRRG	Disruption of the plasma membrane	[216,222,228]
	BAC-CN	Bovine neutrophils	γ-turn	RLCRIVVIRVCR	Disruption of the plasma membrane	[16,155,229]
	VIP	Human neurons (Myenteric and sub-mucosal) and nerve terminals in the GI tract	α-helix	HSDAVFTDNYTRLRKQMAVKKYLNSILN	Disruption of the plasma membrane<br>Alteration intracellular trafficking<br>Disturbance of glycosomes and mito-chondria	[154,216,230]
	Amphomycin	*Streptomyces canus*	Macrocylic ring	FA-DMDDGDG-Dab-VP-Da-Pip	Inhibits the biosynthesis of the glyco-lipid precursor of GPI	[166,231,232]
	Leucinostatin A	*Paecilomyces* spp.	α-helix	MHA-MeP-Dec-HyL-Aib-Aib-L-L-Aib-βA-N(CH_3)	Ionophore<br>Disruption of the plasma membrane	[166,233]
	Leucinostatin B			MHA-MeP-Dec-HyL-Aib-Aib-L-L-Aib-βA-NH(CH_3)$_2$		
	Alamethicin	*Paecilomyces* spp.	α-helix	Ac-Aib-P-Aib-A-Aib-A-Q-Aib-V-Aib-GL-Aib-PV-Aib-Aib-E-Q-Phol	Ionophore<br>Disruption of the plasma membrane	[167,174,234]

	AS-48	*Enterococcus faecalis*	α-helix	MVKENKFSKIFILMALSFLGLALFSASLQFLPIAHMAKFGI PAAVAGTVLNVVEAGGWVTIVSTAVGSGLSLLAAAGRE SIKAYLKKEIKKKGKRAVIAW	Formation of multilamellar vesicles and myelin-like structures Alteration of the nuclear envelope	[169,216,235]
	Mellitin	*Apis mellifera* (Venom glands)	α-helix	GIGAVLKVLTTGLPALISWIKRKRQQ	Alteration of Ca^{2+} homeostasis Disturbance of mitochondria Disorganizing kinetoplast DNA Allteration of metabolism	[16,176,178,216,236]
	SHP-1	Synthetic	α-helix	SDLGAVISLLLWGRQLFA	Rigidity of the plasma membrane Loss of cell motility	[182]
	TOP-10	Synthetic	α-helix	AGYLLGKINLKALAALAKKIL	Interferes with cellular processes (such as enzymatic activities and nucleic acid synthesis)	[180,183]
T. cruzi	Trialysin	*Triatoma infestans* (Salivary glands)	Mixed (α-helix/ β-sheet)	KIKPGKVLDKFGKIVGKVLKQLKKVSAVAVAMKKGA ALLKKMGVKISPLKCEEKTCKSCVIFKITENSFCTIRF MKNIATYLVVAGEINRKSKFEEKLKLGNMPRCVNVE-FIGKVCMKGIEGHAKSSSGQANVNFCLGLVAEKFGV GAKLCGIYANKKVRVKISPQLFPGATSLDGDIVKLDDN GEDATTLDVDEVEID	Disruption of the plasma membrane	[20,216]
	Def1.3	*Triatoma (Meccus) pallidipennis* (Salivary glands)	Mixed (α-helix -β-sheet)	RATCDLLSFESKWFTPNHAACAAHCILLGNRGGH CVGTLCHCRK	Disruption of the plasma membrane	[22,216]
	Cecropin A	*Hyalophora cecropia*	α-helix	KWKLFKKIEKVGQNIRDGIIKAGPAVAVVGQATQIAK	Disruption of the plasma membrane	[186,190,216]
	Def- α-1	Human neutrophils	Mixed (α-helix -β-sheet)	DIPEVVVSLAWDESLAPKHPGSRKNMACY-CRIPACIAGERRYGTCIYQGRLW	Disruption of the plasma membrane Cytoplasmic vacuolization Induction of nuclear and mitochondrial DNA fragmentation Breakage of the flagellar membrane	[195,196,216,237]

	VIP	Human (Myenteric and sub-mucosal neurons and nerve terminals in the GI tract)	α-helix	HSDAVFTDNYTRLRKQMAVKKYLNSILN	Disruption of the plasma membrane	[104,216]
	Mellitin	*Apis mellifera* (Venom gland)	α-helix	GIGAVLKVLTTGLPALISWIKRKRQQ	Disruption of the plasma membrane<br>Kinetoplast and mitochondrial deterioration<br>Alterations of the Ca^{2+} homeostasis<br>Activation of different cell death pathways	[177,202]
	Polybia-CP	*Polybia paulista* (Venom gland)	α-helix	ILGTILGLLKSL	Induction of ROS<br>Mitochondrial dysfunction<br>Apoptosis-like cell death	[201,238]
	MP			IDWLKLGKMVMDVL	Induction of ROS<br>Mitochondrial dysfunction<br>Inhibition of the GAPDH enzyme	[208,216]
	BatxC	*Bothrops atrox* (Venom gland)	α-helix	KRFKKFFKKLKNSVKKRVKKFFRKPRVIGVTFPF	Disruption of the plasma membrane<br>Production of ROS<br>Mitochondrial dysfunction	[203,216]
	Ctn	*C. durissus terrificus* (Venom gland)	α-helix	KRFKKFFKKVKKSVKKRLKKIFKKPMVIGVTIPF		[204,216]
	DS 01	*Phyllomedusa nordestina* (Skin gland)	α-heli	GLWSTIKNVGKEAAIAAGKAALGAL	Disruption of the cell membrane<br>Inhibition of synthesis of proteins, DNA and RNA	[105,106]
	DS 04			GLWSTIKQKGKEAAIAAAKAAGKAALNAASEAL		
	Phyl 7			FLSLIPHAINAVSAIAKHF		
	Phyl 8			FLSLIPTAINAVSALAKHF		
	Figainin 1	*Boana raniceps* (Skin gland)	α-helix	FIGTLIPLALGALTKLFK	Disruption of the cell membrane<br>Inhibition of synthesis of proteins, DNA and RNA	[211,216]
	Figainin 2			FLGAILKIGHALAKTVLPMVTNAFKPKQ		

	Tach	*Tachypleus tridentatus* (Hemocytes)	β-sheet	KWCFRVCYRGICYRRC	Disruption of the plasma membrane	[185,216]
	Hemocyanin	*Penaeus monodon* (Hemolymph)	Mixed (α-helix/ β-sheet)	LVVAVTDGAADAAVDGLHENTEF-NHYGSHGKYPDNRPHGYPLDRKVP	Disruption of the plasma membrane	[103,216]
	Tempz-1	Synthetic	α-helix	FLPLWLWLWRKLK	Chromatin condensation<br>Mitochondrial cristae disorder<br>Kinetoplast disorganization<br>Increase in the number and degeneration of reservosomes	[17]
	DC 1<br>DC 2<br>DC 3	Synthetic	α-helix	FALALKALKKALKKLKKALKKAL<br>FAKKLAKKLKKLAKKLAKLALAL<br>FARRLARRLRRLARRLARLALAL	Disruption of the plasma membrane	[184]

GI: Gastrointestinal, FA: Fatty acid, Dabe: D-eryr/zro-cr,P-diaminobutyric acid, D-Pip: D-pipecolic, MHA: 4-methyl-2-hexenoic acid, MeP: 4-methylprolin, AHMO: 2-amino-6-hydroxy-4-methyl-8-oxodecanoic acid, HyL: threo-β-hydroxyleucine, Aib: 2-aminoisobutyric, β-A: β-alanine, DMPD: N, N-dimethylpropane1,2-diamine, AC: acetyl, Phol: L-phenylalaninol
